# PD117 Proposed Matrix For Efficient Reassessment Of Selective Benefits

**DOI:** 10.1017/S026646232400357X

**Published:** 2025-01-07

**Authors:** Jun Jang, Yoon-Jeong Choi, Myoung-Hwa Kim

## Abstract

**Introduction:**

To reduce the burden of medical expenses on patients, some noncovered medical technologies with proven safety but uncertain therapeutic effectiveness or cost effectiveness are incorporated into the “selective benefit (SB) system” and reassessed regularly to determine reimbursement scope. This study proposes a matrix based on the usage trends of new technologies (NTs) and alternative therapies (ATs) to facilitate efficient reassessment.

**Methods:**

This study investigated the following five indices: (i) replacement of an NT by an AT; (ii) market shares of NTs; (iii) usage trends of NTs; (iv) usage trends of ATs before and after introduction of NTs; and (v) social demand for NTs. These were combined to generate an algorithm-based matrix that classified 139 NTs into 22 cases and five reimbursement scope categories. Health insurance data from 2009 to 2021 were analyzed to investigate market shares and usage trends. Social demand was evaluated using the last assessment results for each NT.

**Results:**

Using the matrix, 139 NTs were classified as follows: (i) switch to an essential benefit (copayment 20%; n=11); (ii) stay as a SB (copayment 50%; n=19); (iii) stay as a SB (copayment 80%; n=30); (iv) stay as a SB (copayment 90%; n=8); and (v) convert to noncovered (copayment 100%; n=40). The remaining 31 with an insufficient analysis period were classified as a SB (copayment 80%) for further analysis. Excluding the latter 31 SBs, 57 of the 108 (53%) were classified as “stay as a SB” categories, suggesting that these technologies need to be monitored further.

**Conclusions:**

The usage trend driven matrix may be useful for efficient reassessment of NTs. For example, NTs that have a high market share and an increasing usage trend and ATs with a decreasing usage trend after SB of an NT can potentially be switched to an essential benefit.

